# Multilevel survival analysis of the age at first birth among women in Ethiopia

**DOI:** 10.3389/frph.2024.1419537

**Published:** 2024-10-09

**Authors:** Nuru Mohammed Hussen, Gezachew Gebeyehu Arega, Abdu Hailu Shibeshi, Getnet Mamo Habtie, Tigabu Hailu Kassa, Kassaye Getaneh Arge

**Affiliations:** Department of Statistics, Samara University, Semera, Ethiopia

**Keywords:** age at first birth, Ethiopia, Demographic and Health Survey, multilevel survival analysis, teenage childbearing

## Abstract

**Introduction:**

The age at first birth refers to the age at which a woman has her first child. It can significantly influence the demographic behavior of women and the general community. Moreover, teenage childbearing is a serious public health and social problem. The main objective of this study was to identify factors associated with age at first birth among women in Ethiopia.

**Methods:**

Secondary data on women were obtained from the 2019 Ethiopia Mini Demographic and Health Survey (EMDHS). These population-based cross-sectional data were downloaded from the Measure Demographic and Health Survey website (http://www.measuredhs.com). The study included a random sample of 8,885 women aged 15–49 years from 305 enumeration areas. A multilevel survival analysis was employed to identify the factors associated with teenage childbearing among women in Ethiopia.

**Results:**

The majority (67.7%) of randomly sampled women were subjected to teenage childbearing. Women being rural dwellers [hazard ratio (HR) = 1.27, 95% CI: 1.05, 1.54]; women from middle-income families (HR = 1.43, 95% CI: 1.18, 1.74); and women from higher-income families (HR = 1.40, 95% CI: 1.15, 1.70) were associated with a higher risk of teenage childbearing. Conversely, contraception method users (HR = 0.87, 95% CI: 0.77, 0.99), Muslims (HR = 0.75, 95% CI: 0.64, 0.89), Orthodoxes (HR = 0.68, 95% CI: 0.57, 0.80), women with secondary education (HR = 0.53, 95% CI: 0.43, 0.65), women with higher education (HR = 0.28 (95% CI: 0.22, 0.37), and the higher age of household head (HR = 0.99, 95% CI: 0.98, 0.99) were associated with a lower risk of teenage childbearing among women in Ethiopia.

**Conclusion:**

Since the median age of women to have their first child was 18 years old, this study strongly suggests that stakeholders at the federal and regional levels must work closely toward enforcing the legal age of marriage and implementing national adolescents’ and youths’ targeted sexual and reproductive health programs.

## Introduction

The age at first birth is the age at which a woman has her first child. It is one of the most important events in a woman's life as it is a clear transition to motherhood and taking the responsibility of childcare (1). The demographic behavior of women and the general community is significantly influenced by a woman's reproductive age (2). Moreover, the lower age at first birth decreases the woman's decision-making power in areas related to her own reproductive health (1).

Teenage childbearing can have long-term consequences for a woman's socioeconomic well-being (3). Indeed, when women begin to give birth, they must give up certain duties and responsibilities that can take a significant amount of time and resources. This can have negative consequences for career development, education, marital stability, wealth, and, most importantly, health. It also has an impact on the types and availability of care available to women and children, as well as social changes, reproductive habits, and economic circumstances (4). Teenage pregnancies due to the premature delivery of the first child during adolescence are also associated with maternal mortality. There are many reasons for studying the dynamics of birth timing and intervals, including understanding overall family size and maternal mortality (5).

Women should consider their age when deciding whether or not to become pregnant, as they can become pregnant and bear a child from puberty to menopause. Teenage mothers are more vulnerable to various health issues, while older mothers are associated with a higher risk of having pregnancy complications. Research suggests that the ideal age at first birth is 30.5 years (6). Adolescents are more susceptible to early sexual intercourse and unwanted pregnancy, especially in developing countries (7). In these countries, early marriage is the major risk factor for teenage pregnancy followed by childbearing, where over 30% of marriages are below the age of 18 years, among which 14% are below the age of 15 (8). Moreover, among the 11% of teen births occurring annually among women aged between 15 and 19 years, 95% take place in developing countries (9).

Although the global total fertility rate decreased from 5.86 in 1950–1955 to 2.04 in 2022, Africa remained the continent with the highest fertility rate, with particularly high levels in sub-Saharan Africa (10). According to the United Nations 2014 report, out of 66 high-fertility countries, 45 (more than 3.2 children per woman) are concentrated in sub-Saharan Africa. Moreover, the adolescent fertility rate has declined in most countries of the world but remains higher in Africa, especially Sub-Saharan Africa (11).

Ethiopia is Africa's second-most populous country next to Nigeria, with an annual population growth rate of 2.57%, a fertility rate of around five children per woman, and a median age of 19 years (12). In 2021, 39.56% of the total population was under the age of 14 years, with 21.1% of the population aged 15–24 years, including 5.6% of women aged 15–19 years, 13% of whom were already mothers or pregnant with their first child (13). Ethiopia has the world's fourth-highest absolute number of women married or in a partnership before the age of 18 years (14). More than 76% of married or in-union females aged 15–19 years and 65% of married or in-union young women aged 20–24 years have never used any kind of birth control (15). Approximately 22.2% of women aged 20–24 years gave birth before reaching the age of 18 (15). To meet the provisions of the national youth policy, which is concerned with the consolidation and integration of youth development programs into all respective government ministries, and other stakeholders, it is indeed necessary to form youth councils, an inter-federal government offices committee, an inter-regional bureaus committee, a consortium of non-governmental entities, and a nationwide youth forum (16, 17). Furthermore, regional health and youth bureaus are not well organized to design, implement, monitor, and evaluate tailored and evidence-based strategies, approaches, and interventions. Furthermore, present national and regional interventions, which are mostly sponsored by development partners, are short-term, piecemeal projects that are skewed toward urban areas (18). In Ethiopia, single-level and multilevel approaches were conducted to analyze the trend, prevalence, and determinants of teenage childbearing using multilevel logistic regression (7, 19–21), multilevel Cox regression (22), and the binary logistic regression model (23). Hence, in relevance to those pieces of literature, this study aimed to introduce a multilevel survival analysis to identify woman-level and enumeration area (EA)-level predictors of teenage childbearing using the data from the 2019 Ethiopia Mini Demographic and Health Survey (EMDHS).

## Methods

### Data source and study design

This paper uses secondary data sets from the 2019 Ethiopia Mini Demographic and Health Survey (EMDHS). The survey data were downloaded from the Measure DHS website (http://www.measuredhs.com) after a reasonable request, and data use permission was obtained from the institutional review board (IRB) of the International Classification of Functioning, Disability and Health (ICF). The study design for the survey was a population-based cross-sectional study design. The survey conducted from 21 March 2019 to 28 June 2019 was the second EMDHS and the fifth DHS implemented in Ethiopia. The Ethiopian Public Health Institute (EPHI) carried out this survey in partnership with the Central Statistical Agency (CSA) and the Federal Ministry of Health (FMoH), with technical assistance from the ICF and financial and technical support from development partners.

### Sampling design

The sampling frame for the 2019 Ethiopia Mini Demographic and Health Survey was a complete list of the 149,093 enumeration areas (EAs) established for the 2019 Ethiopia Population and Housing Census (EPHC), which was performed by the Central Statistical Agency (CSA). The average coverage for an enumeration area was 131 households. For the 2019 EMDHS, a two-stage stratified cluster sampling design was used. Ethiopia is structured administratively into nine nation-states and two administrative cities, which are then stratified into urban and rural except for Addis Ababa, which is entirely urban, to establish 21 sampling strata. To ensure the precision of sample selection, the allocation was done through an equal allocation where 25 EAs were selected from eight regions and each of the three main regions was assigned 35 EAs: Amhara, Oromia, and the Southern Nations, Nationalities, and Peoples’ Region (SNNPR). Among the 305 EAs selected in the primary stage, 93 were urban and 212 were rural, and in the second stage of selection, an equal probability systematic selection was used to select a fixed number of 30 families per cluster from the newly established household listing. A total of 8,885 women of reproductive age (15–49 years) were eligible and interviewed from a nationally representative sample of 9,012 women.

### Operational definitions

#### Age at first birth

This is defined as the length of time obtained by subtracting the birth date of the mother from the birth date of her firstborn child, measured in years.

#### Wealth index

The wealth status of households was assessed using a combination of both urban and rural indicators, depending on the context and the specific characteristics of the household being assessed. In urban areas, wealth status is often measured using indicators such as income, occupation, education level, housing quality, and ownership of durable assets such as cars, electronics, and appliances. These measures are useful in assessing the standard of living and economic well-being of households in urban areas. On the other hand, in rural areas, wealth status is often measured using indicators such as land ownership, livestock ownership, crop yields, and access to basic infrastructure such as water and sanitation facilities. These measures are more relevant to the rural context and help assess the economic status and livelihoods of households in rural areas.

### Variables in the study

In this study, we have considered two types of variables, namely, response and explanatory variables.

### Response variable

The response variable in this study was the age at first birth of women in years, which was created by subtracting the birth date of a firstborn child from the birth date of a mother. It is an event in which a woman gives birth to her first child. Furthermore, teenage childbearing is a serious public health and social problem in which a teenage girl who has not reached legal adulthood, usually between the ages of 13 and 19, becomes pregnant and gives birth, despite regional variations (24). Moreover, the World Health Organization (WHO) defines teenagers as the transition from childhood to adulthood, especially in the age group 10–19 (25). According to this specification, the variable age at first birth may be either the event (teenage childbearing) or censored (when there is no teenage childbearing).

### Explanatory variables

The selection of explanatory variables was theoretically driven and supported by prior research with regard to factors affecting the age at first birth among married women in Ethiopia (7, 19–21). Therefore, the predictor variables used in this study were women's educational level, age of household head, sex of household head, wealth index, contraceptive method usage, husband's education level attained, and religion, which were potential lower-level predictors of women's age at first birth, while residence was considered as a higher-level predictor. The data were managed with SPSS 26 and analyzed using SAS 9.4. The data were weighted to take into account or adjust for disproportionate sampling and non-sampling responses and to increase the representativeness of the sample. Variable selection was made by the purposeful method, while a univariable analysis was made between a response variable and each predictor separately at a 25% level. Subsequently, a multivariable analysis was performed at a 5% level between a response variable and all significant variables in the univariable analysis. The model selection was made using the general Chi-square and Chi-square per degree of freedom due to the presence of nested models.

### Ethical considerations

This study was a secondary data analysis of the Ethiopia Mini Demographic and Health Survey (EMDHS). The institutional review board (IRB) approved the procedures for DHS public-use datasets that do not in any way allow respondents, households, or sample communities to be identified. An ethical statement was obtained from the institutional review board of the International Classification of Functioning (ICF) to use the survey data set for a study entitled “Multilevel survival analysis of the age at first birth among women in Ethiopia.”

### Survival analysis

The analysis of statistical data in which the outcome variable of interest is the time until an event occurs is known as survival analysis. Comparing two or more estimated survival curves is the most frequently used statistical tool in recent clinical research (26). The simplest way of comparing the survival times obtained from two or more groups is with Kaplan–Meier curves and the log-rank test (27).

### The multilevel survival analysis

Although the single-level Cox regression provides us with the effect of several predictors on the time-to-event outcome, due to the hierarchical nature of the data where each married woman nested in an enumeration area, the multilevel approach is recommended over the standard approach (28). Moreover, the survival model extends readily to multilevel models by different scholars, and this section considers an exposition of this model. The hazard function *h*(*t*) is the probability of the event occurring in the time interval *t*, conditional on no earlier occurrence. The hazard is generally modeled with a logistic regression in the following form:

$$\mathrm{logit}(h_{\mathrm{ij}}(t))=h_{0}(t)+\beta_{2j}X_{\mathrm{ij}}+\beta_{3}Z_{j}$$

Where $h_{0}(t)$ is the baseline hazard at time-specific intercepts, $X_{\mathrm{ij}}$ is a women-level predictor in which the regression coefficient varies at different enumeration areas, and $Z_{j}$ is the enumeration area-level predictor.

However, when there are a large number of time periods, the model can be made more parsimonious by modeling the hazard as a smooth function of t as follows:

$$\mathrm{logit}(h_{\mathrm{ij}}(t))=\beta_{0}+\beta_{1}t_{\mathrm{ijt}}+\beta_{2}X_{\mathrm{ij}}+\;\beta_{3}Z_{j}+U_{0j}+U_{1j}$$

where $U_{0j}\;\mathrm{and}\;\;U_{1j}$ are the intercept and slope variations, respectively.

## Results

### Descriptive statistics

This study was conducted on the factors of the age at first birth among 8,885 randomly selected women for the 2019 Ethiopia Mini Demographic and Health Survey. Accordingly, among the randomly sampled women in the country, the majority (67.7%) of them were subjected to teenage childbearing, whereas the remaining 32.3% were censored. The median age at which they had their first child was 18 years, which falls into the category of teenagers and implies that the probability that the randomly selected women will survive without giving birth for 18 years or more is 0.5. However, among these women, the majority (67.8%) belong to rural residents, while the remaining 32.2% are urban residents of Ethiopia. Most of the women (71.2%) did not use any methods of contraception, while only 28.8% of them used any different methods. In addition, when compared to other corresponding labels of a factor, the majority of these sample women (41.7%) attended primary school, were Orthodox (41.5%), were male-headed (79.3%), and had the highest income level (25.7%). Moreover, the significance of the Chi-square value showed that there is a significant association between the teenage status of randomly selected women and categorical predictors at a 5% level of significance (Table 1).

**Table 1:** Teenage childbearing status and its sociodemographic and economic characteristics.

Variable	Categories	Frequency	Percent	*P*-value^a^
Teen birth status	Teen birth	3,963	67.7	
	No teen birth	1,892	32.3	
Place of residence	Urban	2,861	32.2	0.00
	Rural	6,024	67.8	
Contraception method	Not used	6,329	71.2	0.00
	used	2,556	28.8	
Women's educational level	No education	3,589	40.4	
	Primary	3,701	41.7	0.00
	Secondary	1,088	12.2	
	Higher	507	5.7	
Religion	Orthodox	3,685	41.5	0.00
	Catholic	47	0.5	
	Protestant	2,435	27.4	
	Muslim	2,619	29.5	
	Others	98	1.1	
Wealth index	Poorest	1,437	16.2	.00
	Poorer	1,615	18.2	
	Middle	1,671	18.8	
	Richer	1,874	21.1	
	Richest	2,287	25.7	
Sex of household head	Male	7,050	79.3	0.00
	Female	1,835	20.7	

^a^ The results are based on the Chi-square test.

### Model selection

Model selection is the task of selecting the statistically best model that fits the statistical data among a series of candidate models. The intra-class correlation was computed from the intercept-only model for the purpose of identifying the proportion of variance explained by the grouping structure. Then the value demonstrated the use of multilevel analysis rather than single-level analysis by ignoring such grouping structures. Moreover, due to the hierarchical nature of the data, the presence of predictors at different levels in multilevel studies makes the model selection different from standard methods. Accordingly, different mixed-effects models were considered to analyze the age at first birth of randomly selected women in Ethiopia. Because the Chi-square per degree of freedom approaches one and the addition of a random slope has no effect on the fit statistics, the random intercept model with fixed woman-level and enumeration area-level predictors was the best fit for the data. Although it is difficult to make comparisons among nested modes using the log residual pseudo-likelihood statistic, it has the smallest value for this model (Table 2).

**Table 2:** Multilevel model selection.

Fit statistics	Model			
	The intercept-only model (M0)	M0 + level 1 predictors (M1)	M1 + level 2 predictors (M2)	M2 + random slope
Res log pseudo-likelihood	−12,647.92	−12,908.48	−12,914.64	−12,914.64
Generalized Chi-square	2,649.96	5,598.01	5,730.42	5,730.42
General Chi-square/DF	0.45	0.96	0.98	0.98

### The intercept-only model

A model fitted without any predictor variables is termed the “intercept-only model.” In multilevel analysis, the random effect results from the intercept-only model are important to indicate the necessity of such analysis. The residual error variance at the women's and enumeration area levels was estimated to be 0.45 and 2.42, respectively, as a result of the maximum mean pseudo-likelihood estimation. We can conclude that there is more variation among the different enumeration areas (2.42) than within the enumeration areas (0.45). This will be discussed further when we calculate the intra-class correlation (ρ) for this model below. From the table below, we can calculate the proportion of variance explained by the grouping structure by using the intra-class correlation (ρ), which is given as follows:

$$\rho =\frac{\sigma^{2}u_{0}}{\sigma^{2}\varepsilon_{0}+\sigma^{2}\;u_{0}}\;=\frac{2.42}{2.42+0.45}=0.84$$

This result implies that 84% of the variation in women's age at first birth is at the enumeration level and the remaining 16% is between women (Table 3).

**Table 3:** Parameter estimation for the intercept-only model.

Estimates for fixed effects							
Effect	$\hat{\beta }$ [Exp($\hat{\beta }$)]	SE	*T* value	Pr > |t|	Alpha	Lower	Upper
Intercept	0.64 (1.90)	0.03	23.40	<.00	0.05	0.59	0.70
Estimates for random effects							
Covariance parameter	Estimate		SE				
Intercept (*U*_oj_)	2.42		0.08				
Residual (*e*_ij_)	0.45		0.05				

### Final two-level survival analysis

Since all the predictors were significant at a 25% level in the univariable analysis, multivariable analysis was conducted by using all the potential predictors in the study. The age of the household head, contraceptive method usage, residence, educational level of women, wealth index, and religion were statistically significant predictors for the age at first birth among married women in Ethiopia. The estimates from the random effect result revealed that the variation in the age at first birth was higher (0.98) between the different enumeration areas than within the enumeration areas (0.09) (Table 4).

**Table 4:** Parameter estimation for the two-level survival model.

Estimates for fixed effects					
				95% CI	
Variable	Categories	$\hat{\beta }$ [exp (*β*)]	SE	Lower	Upper
Intercept		1.39 (4.01)	0.16	2.92	5.52
Contraception use (Ref = not used)	Used	−0.14 (0.87)	0.06	0.77	0.99
Residence (Ref = urban)	Rural	0.24 (1.27)	0.10	1.05	1.54
Wealth index (Ref = poorest)	Poorer	0.16 (1.18)	0.09	0.98	1.42
	Middle	0.36 (1.43)	0.099	1.18	1.74
	Richer	0.34 (1.40)	0.10	1.15	1.70
	Richest	−0.15 (0.86)	0.12	0.68	1.08
Religion (Ref = protestant)	Catholic	0.47 (1.60)	0.35	0.81	3.16
	Muslin	−0.29 (0.75)	0.08	0.64	0.89
	Orthodox	−0.39 (0.68)	0.08	0.57	0.80
	Other	0.23 (1.26)	0.31	0.69	2.30
Education level (Ref = illiterate)	Primary	0.05 (1.05)	0.07	0.91	1.20
	Secondary	−0.64 (0.53)	0.10	0.43	0.65
	Higher	−1.26 (0.28)	0.14	0.22	0.37
Age of household head		−0.01 (0.99)	0.00	0.98	0.99
Sex of household head (Ref = male)	Female	−0.05 (0.95)	0.07	0.83	1.08
Estimates for random effects					
Covariance parameter		Estimate		SE	
Intercept (*U*_oj_)		0.98		0.01	
Residual (*e*_ij_)		0.09		0.01	

Key: exp (*β*) = adjusted hazard ratio (HR).

## Discussion

This study was conducted based on secondary data obtained from randomly selected women across the country and found that the majority of the women (67.7%) were subjected to teenage childbearing. This proportion is much higher than the findings reported from Kenya (38.9%) (29), Nigeria (19.0%) (30), Uganda (25%) (31), Slovakia (17%) (32), and Canada (2.9%) (33). This might be due to the differences in the demographic and sociocultural characteristics of the countries, such as women's educational attainment, marital age, use of contraception, and individual wealth status.

The estimated hazard ratio (HR) for the age of the household head was 0.99, implying that a 1-year increase in the age of the household head reduced the risk of teenage childbearing among women by 1% while all other variables remained constant. The result was consistent with the study by Uwizeye et al. (34), where increasing the age of the household head is associated with a lower risk of teenage childbearing.

The estimated hazard ratio of teenage childbearing among rural women compared to urban women was 1.27, indicating that the hazard of teenage childbearing among rural women was 27% higher than the hazard of teenage childbearing among urban women, keeping all other variables constant. The result was consistent with other studies (7, 21, 35), which reported that rural women are more vulnerable to the risk of teenage childbearing than urban women. This could be due to the lack of access to sexual and reproductive health services and knowledge and materials for early pregnancy prevention in rural areas of Ethiopia.

The estimated hazard ratio of teenage childbearing among women who have used contraception methods compared to those who did not indicated that the risk of teenage childbearing among women who have used contraception methods was 13% less than the hazard of teenage childbearing among women who have not used any method, keeping all other variables constant. The result was consistent with other studies (7, 21, 35), which also reported that the lower contraception use rate within the community was substantially associated with the higher risk of teenage childbearing.

The risk of teenage childbearing among women with middle and richer income levels was 43% and 40% respectively, higher than the risk of teenage childbearing among women with the poorest income levels, keeping all other variables constant. The findings were consistent with a study (7) that reported that women from lower-income families were more vulnerable to teenage childbearing than those from middle- and higher-income families. Moreover, the studies conducted in the United States of America and East Africa showed how teens from low-income families engage in sexual activities to generate income (36–40).

Keeping all other variables constant, the risk of teenage childbearing among Orthodox and Muslim women was 32% and 25%, respectively, lower than the risk of teenage childbearing among protestant women. The result was consistent with other studies (41–43), where religious aspects such as prohibitions, affiliations, not having sex, infrequent sex, attitudes, norms, and beliefs toward birth control have a significant influence on teens’ decisions to get pregnant.

The risk of teenage childbearing among women enrolled in secondary and higher education was 0.53 and 0.28, respectively, which indicates that women with secondary and higher education were less likely to have teenage childbearing than illiterate women. This may be due to the difference in information sharing and the change in attitude and knowledge about early pregnancy and family planning. More specifically, the risk of teenage childbearing among women enrolled in secondary and higher education was 47% and 72%, respectively, less than the risk of teenage childbearing among illiterate women, keeping all other variables constant. The result was consistent with the studies conducted in Bangladesh and Kenya (7, 44, 45), which reported that teenagers with secondary and higher education were 60%–70% less likely to have transitioned to their first pregnancy at each age compared to those with primary or lower education.

### Strengths and weaknesses of the study

The strength of this study is the use of the Demographic and Health Survey (DHS) data. The survey uses equal probability systematic sampling for selecting women from different geographical territories so that representative and quality data can be obtained about women at household and community levels. Findings from this study can be generalized to the study population. On the other hand, the study does not contain predictors such as age at first marriage, which could be important to the study.

## Conclusion

This study can be concluded to have identified some factors associated with the age at first birth among women in Ethiopia using the framework of multilevel survival analysis on the 2019 Ethiopia Mini Demographic and Health Survey data. The median age at which the women had their first child was 18 years, indicating that the risk of teenage childbearing is high in the country and that each woman must be closely monitored. As the result revealed, there was more variation in women's age at first birth at the enumeration level, so analysis of such data using a single-level approach may ignore such variation. Moreover, the age of the household head, contraceptive method usage, residence, educational level of women, wealth index, and religion were found to be the predictors of the age at first birth of women in Ethiopia. These findings have valuable policy implications for intervention and program design, especially, since stakeholders at the federal and regional levels must work jointly towards enforcing the legal age of marriage and implementing national adolescents’ and youths’ targeted sexual and reproductive health programs.

## Data Availability

The datasets presented in this study can be found in online repositories. The names of the repository/repositories and accession number(s) can be found in the article/Supplementary Material.
